# Postoperative ischemia and neurological deficits after glioma resection: A systematic review and meta-analysis

**DOI:** 10.1093/nop/npaf122

**Published:** 2025-12-16

**Authors:** Arthur T J van der Boog, Eva E van Grinsven, Matea Rados, Tom J Snijders, Joost J C Verhoeff, Pierre A Robe

**Affiliations:** Department of Neurology and Neurosurgery, UMC Utrecht Brain Center, University Medical Center Utrecht, Utrecht University, Utrecht, The Netherlands; Department of Radiotherapy, University Medical Center Utrecht, Utrecht University, Utrecht, The Netherlands; Department of Neurology and Neurosurgery, UMC Utrecht Brain Center, University Medical Center Utrecht, Utrecht University, Utrecht, The Netherlands; Department of Neurology and Neurosurgery, UMC Utrecht Brain Center, University Medical Center Utrecht, Utrecht University, Utrecht, The Netherlands; Department of Neurology and Neurosurgery, UMC Utrecht Brain Center, University Medical Center Utrecht, Utrecht University, Utrecht, The Netherlands; Department of Radiotherapy, University Medical Center Utrecht, Utrecht University, Utrecht, The Netherlands; Department of Radiotherapy, Amsterdam University Medical Center, Vrije Universiteit, Amsterdam, The Netherlands; Department of Neurology and Neurosurgery, UMC Utrecht Brain Center, University Medical Center Utrecht, Utrecht University, Utrecht, The Netherlands

**Keywords:** glioma, neurological deficits, postoperative ischemia, risk factors

## Abstract

**Background:**

Postoperative ischemia is a known complication of glioma resection that is registered by diffusion-weighted MRI (DWI) and can lead to new neurological deficits. The aim of this review was to assess the incidence of ischemia after glioma resection and identify potential risk factors and consequences.

**Methods:**

A systematic review was conducted according to the PRISMA guidelines. Cohort studies reporting surgeries for WHO grade II-IV gliomas followed by postoperative DWI within 72 h were included. Pooled, weighted incidences for postoperative ischemia and neurological deficits were calculated with a binary random-effects model.

**Results:**

In total, 29 studies were included in this review, of which 25 studies with 2729 cases were included for meta-analysis. Pooled incidence was 48% (95% confidence interval [CI]: 36%-59%) for postoperative ischemia and 32% (95% CI: 23%-41%) for postoperative transient and persistent neurological deficits. Postoperative ischemia with any neurological deficits occurred in 15% (95% CI: 10%-20%) of patients, postoperative ischemia without neurological sequelae in 26% (95% CI: 17%-35%), and 41% (95% CI: 30%-52%) of patients with postoperative ischemia experienced neurological deterioration. Postoperative ischemia was associated with opercular, insular, or temporal tumor locations, proximity to and infiltration of perforating arteries, prior radiotherapy, age and intraoperative hemodynamic decline, among others. Postoperative ischemia was related to neurological deterioration, recurrence, and overall survival in some studies.

**Conclusion:**

Postoperative ischemia is a quite frequent complication of glioma resection affecting functional outcome and disease progression. Potential risk factors may give insight in preventive measures and future studies with precise spatial analyses could identify vulnerable brain regions.

Key Points

- This is a comprehensive systematic review on the frequency of postoperative ischemia and subsequent neurological deficits after glioma resection.
- Potential risk factors are reported that might provide strategies for prevention and future research.

Importance of the Study

This systematic review of PubMed, Embase, and Cochrane Library presents a comprehensive meta-analysis examining the occurrence of postoperative ischemia and subsequent neurological deficits in patients with a grade II-IV glioma. A total of 29 clinical studies were included that reported on acute ischemia identified at early postoperative diffusion-weighted imaging within 72 h. The calculated pooled incidences revealed that ischemia is a frequent, yet inconsistently reported complication following glioma resection and is associated with transient and persistent neurological deficits. Additionally, this review highlights that postoperative ischemia is linked to disease control and overall survival and identifies several potential clinical, radiological, and intra-operative risk factors. A deeper understanding of the causes, course, and consequences of ischemia after glioma resection could inform preventive strategies, guide surgical decision-making and patient counseling, and pave the way for future research studies.

Surgery is often the first step in the treatment of cerebral gliomas. Surgical risks should be balanced against benefits like obtaining histological diagnosis, relief of symptoms by mass decompression, or improving survival. In addition to the surgical risks, including wound infection, wound-healing problems, and possible cerebrospinal fluid leaks,^1^ surgery for brain tumors also comes with the risk of developing temporary and lasting neurological deficits.^2^ Postoperative neurological deficits can arise by direct surgical damage to functional brain tissue or perioperative tissue retraction, postoperative epilepsy, or edema. Additionally, focal ischemia through vascular damage or vasospasm can play a part in the development of postoperative neurological deficits.^3^ Postoperative ischemia could explain new postoperative deficits after surgery in cases for which there were no evident intraoperative causes. However, there is no consensus on the frequency of this treatment complication, nor of its causes or risk factors.

The aim of this review was to explore the incidence and risk factors of ischemia after the resection of WHO grade II-IV supratentorial gliomas and its relation to incidence of new or worsened postoperative neurological deficits.

## Methods

To define the incidence and risk factors of ischemia, we searched for English and Dutch cohort studies reporting surgeries for WHO grade II-IV gliomas followed by diffusion-weighted imaging (DWI) within 72 h. In order to retrieve suitable studies, a systematic literature search of PubMed, Embase, and Cochrane Library was conducted according to the Preferred Reporting Items for Systematic Reviews and Meta-Analyses (PRISMA) guidelines.^4^ The first author designed the search strategy in consultation with a librarian and used a combination of synonyms for “neurosurgery,” “glioma,” and “ischemia” as per the review protocol (Supplementary 1). These keywords were searched in title and abstract from articles published up until July 1, 2024. Deduplication was performed in EndNote version 21.5 according to the method by Bramer et al.^5^

Two authors independently screened the articles by title and abstract for their documentation of ischemia on postoperative magnetic resonance imaging (MRI) in patients with a glioma. Nonclinical papers, reviews, conference papers, and other articles that obviously did not meet the inclusion criteria were excluded in this manner. Included articles were subsequently screened by full text. Articles not reporting use of DWI within 72 h after surgery were excluded. Furthermore, articles including other cerebral lesions were excluded, unless the results of supratentorial WHO grade II-IV gliomas were documented separately. Although brain tumor classification has evolved, with an increasing role of molecular markers, we followed the WHO classification system from the original study. As most included studies preceded the introduction of the WHO 2021 classification,^6^ we used the older WHO II-IV classification throughout this paper, rather than the contemporary WHO 2-4 system. Finally, included articles were screened for suspected overlap in datasets, based on treatment center and timeframe of inclusion. In case of suspected overlap for the majority of patients, the studies with the largest sample size were included in the meta-analysis. The excluded studies were utilized for interpretation of risk factors for postoperative ischemia and neurological deficits. A consensus meeting was held to resolve any disagreements.

Data extraction was performed by the first author and included study characteristics, design, sample size, tumor characteristics, presence and volume of postoperative ischemia, and presence of new postoperative focal neurological deficits. Nonfocal clinical states such as delirium and disorientation were excluded where possible. Subsequently, the articles were analyzed for risk factors for either postoperative ischemia or neurological deficits.

The first author performed quality assessment by means of the Newcastle-Ottowa Scale (NOS),^7^ modified to be applicable for single-arm studies. In this modification, the comparability domain was omitted as well as one item of the selection domain concerning the nonexposed cohort.^8^ Instead of 9, a maximum of 6 points could be earned per study in the modified NOS. Scores of 5 and 6 points were considered to be a low risk of bias, 3 and 4 points a moderate risk of bias, and 2 or lower a high risk of bias.

Finally, a meta-analysis was conducted with Open Meta Analyst.^9^ Heterogeneity was assessed using the I^2^ measure and Cochran’s Q test. A binary random-effects model with DerSimonian estimator for between-study variance was used to calculate pooled, weighted incidences of postoperative ischemia and neurological deficits. Studies that did not report secondary outcomes were omitted from analyses of those outcomes.

## Results

### Search Results

**Figure 1.**
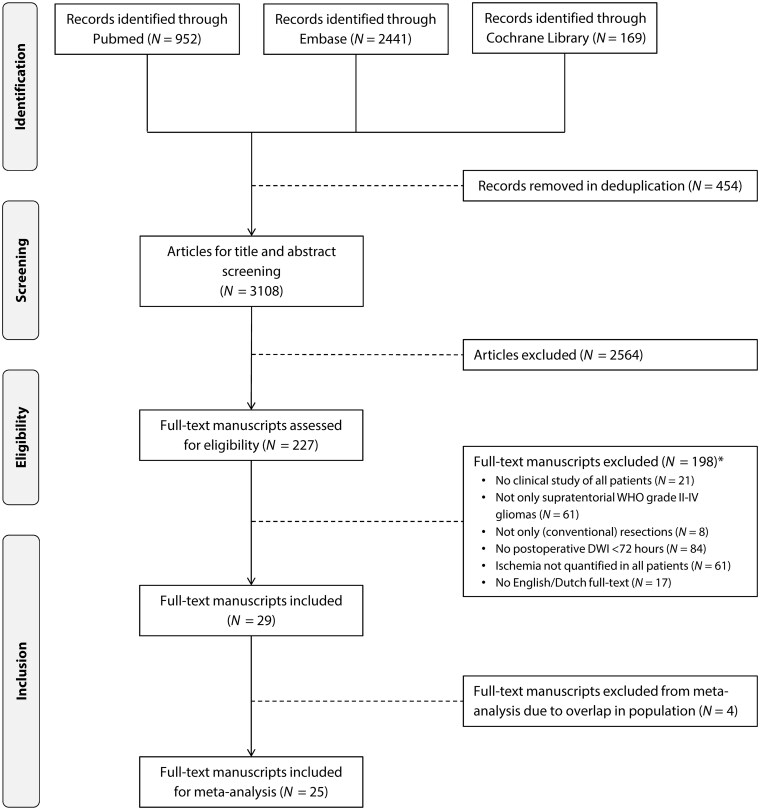
Search strategy. *The sum of the reasons for exclusion exceeds the number of studies excluded, as some had multiple reasons for exclusion.

The search criteria yielded 3108 results after deduplication. After screening, 4 prospective^10-13^ and 25 retrospective^14-38^ studies were included (Figure 1). The largest of 3 studies with overlapping cohorts between May 2008 and March 2015 by Bette et al.was included in meta-analysis.^14-16^ The study by Gempt et al.was included in meta-analysis despite a potential overlap of glioblastoma (GBM) patients with Bette et al.between January 2009 and December 2010.^15^^,^^29^ The studies by Thiepold et al.and Dützmann et al.were included as well, despite a potential overlap of GBM patients between January 2008 and October 2010.^20^^,^^34^ Since the study by Madonnet had an expected overlap of 50% with the study of Loit et al. only the latter was included in meta-analysis.^30^^,^^31^ As both studies of Strand et al.reported on overlapping cohorts, only one was included in meta-analysis.^12^^,^^22^ Finally, 3 studies by Smith et al. Pirzkal et al. and Magill et al.had a potential overlap based on study center.^11^^,^^17^^,^^38^ However, since years of publication and inclusion criteria differed sufficiently, all studies were included in the meta-analysis.

### Study Characteristics

As per the inclusion criteria 29 studies reporting 3049 cases were included (Table 1). After excluding overlapping studies, a total of 2729 cases were reported in 25 studies included for meta-analysis. Some articles reported the presence of neurological deficits in addition to the presence of ischemia. Information on neurological deficits were described in 13 studies. All results regarding postoperative ischemia and neurological deficits can be found in Table 2, and potential risk factors in Table 3. Quality assessment was performed for all studies in this review. Six studies^20^^,^^21^^,^^24^^,^^26^^,^^31^^,^^38^ had a moderate risk of bias and 23 studies^10-19^^,^^22^^,^^23^^,^^25^^,^^27-30^^,^^32-37^ showed a low risk of bias (Supplementary 2).

**Table 1.** Baseline table of all included studies

Study	Study design	Population size (n)	Tumor	Tumor location	Tumor volume (cm^3^)	MRI field strength (Tesla)	Time to DWI (h)	Recurrence (n)	Prior radiotherapy
Smith (2005)^11^	Prospective	44	Glioma WHO grade II-IV	All lobes	··	1.5	<48	··	··
Ulmer (2006)^32^	Retrospective	50	GBM	Unspecified	··	1.5	<72	··	··
Kumabe (2007)^35^	Retrospective	8	Glioma WHO grade II-IV	Operculum (around orofacial cortices)	··	1.5	<72	··	··
Pirzkal (2009)^17^	Retrospective	32	GBM	Unspecified	··	1.5	Immediately	0	0
Dützmann (2012)^34^	Retrospective	177	Glioma WHO grade II-IV	All lobes	··	1.5	<72	47	··
Gempt (2013)^29^	Retrospective	70	Glioma WHO grade II-IV	All lobes	··	3	<48	19	10
Farace (2013)^19^	Retrospective	37	GBM	Unspecified	··	1	<72	··	··
Pamir (2013)^10^	Prospective	14	Glioma WHO grade II	All lobes	··	3	24-48	··	··
Thiepold (2015)^20^	Retrospective	165	GBM	Unspecified	··	3	<72	··	··
Bette (2016)^15^	Retrospective	251	GBM	Unspecified	30.7	3	Unspecified	0	1
Majós (2016)^28^	Retrospective	60	GBM	Unspecified	··	1.5	<72	··	··
Bette (2017)^16^	Retrospective	179	GBM	Unspecified	24.6	3	Unspecified	44	42
Bette (2018)^14^	Retrospective	129	GBM	Unspecified	29.6	3	<72	0	0
Magill (2018)^38^	Retrospective	52	Glioma WHO grade II-IV	Primary motor cortex	··	Unspecified	<48h	4	··
Loit (2019)^30^	Retrospective	115	Glioma WHO grade II-IV	All lobes	··	1.5/3	<48	··	··
Mandonnet (2019)^31^	Retrospective	12	IDH-mutated glioma	Insula	68.5	Unspecified	Immediately	0	0
Przybylowski (2020)^36^	Retrospective	100	Glioma WHO grade II-IV	Insula	62.4	Unspecified	<48	18	··
White (2019)^18^	Retrospective	23	High-grade glioma	Unspecified	··	1.5/3	<24	··	··
Bø (2020)^33^	Retrospective	47	Glioma WHO grade II	All lobes	23.2	1.5/3	<72	0	0
Zetterling (2020)^13^	Prospective	93	Glioma WHO grade II-IV	All lobes	··	Unspecified	<48	··	··
Rossi (2021)^37^	Retrospective	95	Glioma WHO grade II-IV	Insula	71.1	3	<48	45	··
Rosenstock (2021)^21^	Retrospective	66	Glioma WHO grade II-IV	Motor eloquent regions	23.4	3	<72	22	··
Strand (2021)^12^	Prospective	539	Glioma WHO grade II-IV	All lobes	··	Unspecified	<72	219	140
Berger (2022)^23^	Retrospective	239	High-grade glioma	All lobes	32.8	Unspecified	<48	86	69
Hou (2022)^26^	Retrospective	75	Glioma WHO grade II-IV	Insula	57.3	Unspecified	<72	2	0
Strand (2022)^22^	Retrospective	175	Glioma WHO grade II-IV	All lobes	··	Unspecified	<72	58	··
van der Boog (2023)^27^	Retrospective	144	Glioma WHO grade II-IV	Unspecified	33.2	1.5	<72	36	28
Morshed (2024)^24^	Retrospective	151	Glioma WHO grade II-IV	Perirolandic	··	Unspecified	<48	68	··
Biswas (2024)^25^	Retrospective	66	Glioma WHO grade II-IV	Insula	92.6	Unspecified	<48	14	4

Abbreviations: DWI, diffusion-weighted imaging; GBM, glioblastoma.

**Table 2.** Incidence of postoperative ischemia and neurological deficits in the studies included in meta-analysis

Study	Population size (n) included in meta-analysis	Postoperative ischemia on DWI		Ischemia volume (cm^3^)	Postoperative neurological deficits (n)			Combined ischemia and neurological deficits (n)		
		*n*	*%*		*Total*	*Transient*	*Persisting*	*Total*	*Transient*	*Persisting*
Smith (2005)^11^	44	28	63.6	··	··	··	··	··	··	··
Ulmer (2006)^32^	50	35	70.0	14.9	9	··	··	5	··	··
Kumabe (2007)^35^	8	8	100.0	··	6	2	4	6	2	4
Pirzkal (2009)^17^	32	21	65.6	··	··	··	··	··	··	··
Dützmann (2012)^34^	177	46	26.0	··	··	··	··	9	··	··
Gempt (2013)^29^	70	32	45.7	··	23	13	10	16	8	8
Farace (2013)^19^	37	11	29.7	··	··	··	··	··	··	··
Pamir (2013)^10^	14	5	35.7	··	··	··	··	··	··	··
Thiepold (2015)^20^	165	46	27.9	17.1	··	··	··	··	··	··
Bette (2016)^15^	251	226	90.0	2.0	··	··	··	··	··	··
Majós (2016)^28^	60	26	43.3	··	··	··	··	··	··	··
Magill (2018)^38^	52	36	69.2	··	··	··	20	··	··	17
Loit (2019)^30^	115	52	45.2	1.6	··	··	0*	··	··	0^a^
Przybylowski (2020)^36^	100	23	23.0	··	18	8	10	··	··	··
White (2019)^18^	23	19	82.6	··	··	··	··	··	··	··
Bø (2020)^33^	47	29	61.7	··	14	10	4	9	7	2
Zetterling (2020)^13^	93	12	12.9	··	39	33	6	9	8	1
Rossi (2021)^37^	95	19	20.0	··	52	47	5	11	··	··
Rosenstock (2021)^21^	66	20	30.3	··	11	··	9^b^	··	··	··
Strand (2021)^12^	539	238	44.2	1.7	··	··	··	··	··	··
Berger (2022)^23^	239	30	12.6	13.2	··	··	··	13	··	··
Hou (2022)^26^	75	44	58.7	7.8	9^c^	2	7	9	2	7
van der Boog (2023)^27^	144	93	64.6	3.5	46	11	27^d^	36	8	22^d^
Morshed (2024)^24^	158	57	35.6	··	··	··	5	··	··	3
Biswas (2024)^25^	66	30	45.5	··	30	20	7^e^	16	10	6

Abbreviation: DWI, diffusion-weighted imaging.

a Only motor and language function, visual field defects were not taken into account.

b 61 patients available for 3-month follow-up.

c Only motor deficits taken into account.

d 129 patients available for 3-month follow-up; 2 deficits were identified at 3-month follow-up that were not reported at discharge.

e 27 patients available for 6-month follow-up.

**Table 3.** Risk factors for postoperative ischemia and neurological deficits

Study	Risk factors for postoperative ischemia	Risk factors for postoperative neurological deficits
**Kumabe (2007)^35^**	Opercular tumor location	··
	Posterior insular tumor location	
**Dützmann (2012)^34^**	Opercular tumor location	··
	Insular tumor location	
	Temporal tumor location	
**Gempt (2013)^29^**	Proximity to central arteries	Ischemia
	Prior radiotherapy	
**Bette (2016)^15^**	Mean perioperative diastolic blood pressure^a^	··
	Perioperative arterial pressure^a^	
	Length of surgery^a^	
	Fluid balance^a^	
**Magill (2018)^38^**	Awake surgical resection	Ischemia
**Przybylowski (2020)^36^**	··	High-grade glioma
**Bø (2020)^33^**	··	Ischemia
**Zetterling (2020)^13^**	··	Preoperative deficits
		Eloquent tumor location
**Rossi (2021)^37^**	Tumor encapsulation of deep perforating arteries	Tumor encapsulation of deep perforating arteries
**Rosenstock (2021)^21^**	··	Motor cortex infiltration
		nTMS risk factors
		Lower FA and higher ADC values
		Irreversible MEP amplitude decrease
**Strand (2021)^12^**	Temporal tumor location	··
	Cerebrovascular disease^b^	
	Tumor volume^a^	
	Intra-operative bleeding^a^	
	Age	
**Berger (2022)^23^**	Insular tumor location	Ischemia
	Temporal tumor location	
	Preoperative high level of platelets	
	Per-operative language decline	
**Hou (2022)^26^**	MEP amplitude decline	Flat inner edge tumor sign
	Flat inner edge tumor sign	MEP amplitude decline
**Strand (2022)^22^**	Surgeon-reported vein sacrifice	··
	Use of ultrasonic aspirator^b^	
**van der Boog (2023)^27^**	Insular tumor involvement	Confluent ischemia
	No temporal tumor involvement	Preoperative dexamethasone
	Use of intraoperative vasopression	Intraoperative hypocalcemia^c^
**Morshed (2024)^24^**	··	Insular tumor location
		Preoperative deficits
		Ischemia^d^
		Intra-operative MEP changes
**Biswas (2024)^25^**	··	Dominant lobe
		Fronto-lateral opercular tumor location
		Pariëtal opercular tumor location
		Asleep surgery^e^
		Ischemia^d^
		MEP changes^d^

Abbreviations: ADC, apparent diffusion coefficient; FA, fractional anisotropy; MEP, motor evoked potential; nTMS, navigated transcranial magnetic stimulation.

a Related to volume of postoperative ischemia.

b Related to sector-shaped infarction.

c Related to severe deficits.

d Related to prolonged and/or persistent deficits.

e Related to transient deficits.

### Postoperative Ischemia

**Figure 2.**
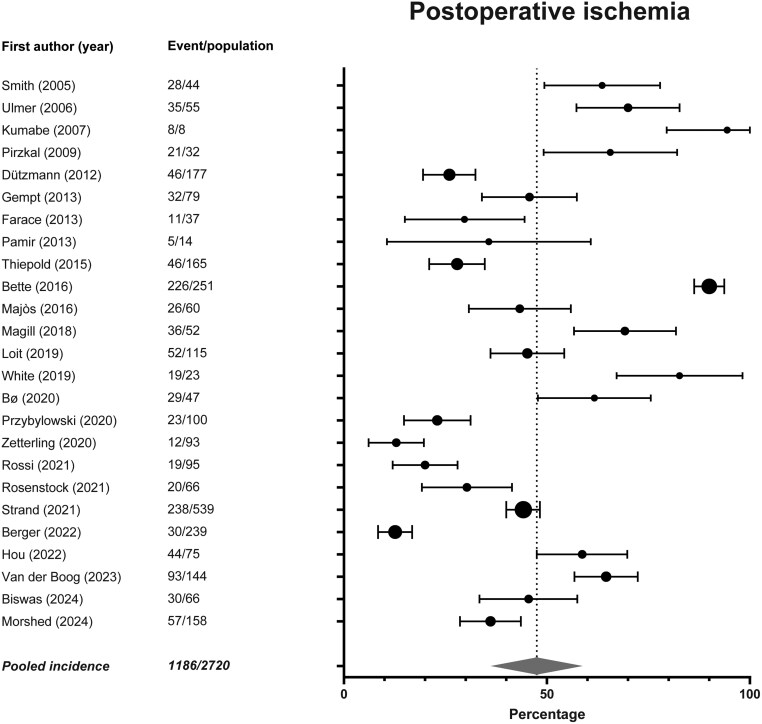
Forest plot and meta-analysis of incidence of postoperative ischemia. Overall incidence of symptomatic and asymptomatic postoperative ischemia on DWI is 43.6%, ranging from 12.5% to 100.0% in the studies. Pooled, weighted incidence is 47.5% (95% confidence interval: 36.2%-58.8%), I^2^: 97.94% (*P* < .001).

All 25 studies in the meta-analysis reported the incidence of postoperative ischemia on DWI. Incidence of postoperative ischemia varied between 13% and 100%. In studies that focused on specific tumor locations, the incidence of postoperative ischemia was 100% (8/8) for the opercular region, 30% (20/66) and 69% (36/52) for motor eloquent regions, 23% (23/100) to 59% (44/75) for the insula, and 36% (57/158) for the perirolandic area, respectively.^21^^,^^24-26^^,^^35-38^ Meta-analysis on the incidence of ischemia showed that the pooled incidence of any form of postoperative ischemia is 48% (95% confidence interval [CI]: 36%-59%) (Figure 2).

### Postoperative Neurological Deficits

**Figure 3.**
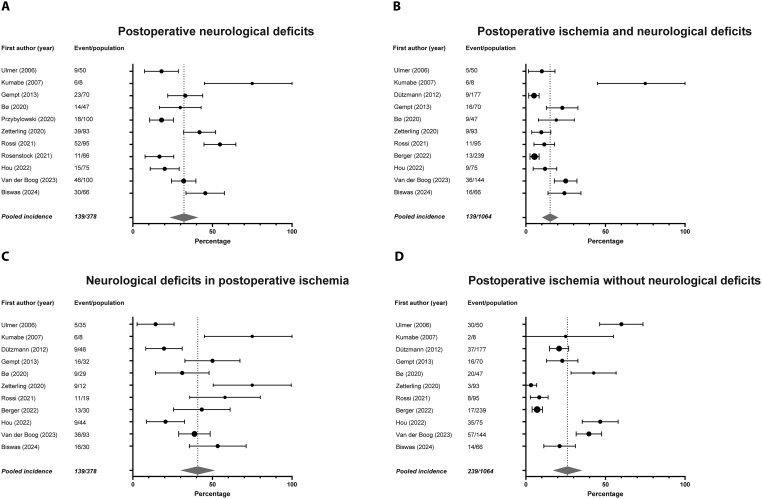
Forest plots and meta-analyses. (A) Incidence of postoperative neurological deficits. Overall incidence of transient and persisting postoperative neurological deficits is 31.8%, ranging from 16.7% to 75.0% in the studies. Pooled, weighted incidence is 32.1% (95% confidence interval: 23.1%-41.1%), I^2^: 88.72% (*P* < .001). (B) Incidence of combined postoperative ischemia and neurological deficits. Overall incidence of both postoperative ischemia on DWI and neurological deficits is 13.1%, ranging from 5.1% to 75.0% in the studies. Pooled, weighted incidence is 15.2% (95% confidence interval: 10.1%-20.4%), I^2^: 85.73% (*P* < .001). (C) Incidence of neurological deficits in patients with postoperative ischemia. Overall incidence of neurological deficits in patients with postoperative ischemia on DWI is 36.8%, ranging from 14.3% to 75.0% in the studies. Pooled, weighted incidence is 40.8% (95% confidence interval: 29.9%-51.7%), I^2^: 81.61% (*P* < .001). (D) Incidence of postoperative ischemia without neurological deficits. Overall incidence of asymptomatic postoperative ischemia on DWI is 22.5%, ranging from 3.2% to 60.0% in the studies. Pooled, weighted incidence is 26.1% (95% confidence interval: 16.9%-35.4%), I^2^: 94.92% (*P* < .001).

The presence of any postoperative neurological deficits was reported in 11 studies in meta-analysis, including 814 patients.^13^^,^^21^^,^^25-27^^,^^29^^,^^32^^,^^33^^,^^35-37^ The incidence of postoperative neurological deficits in the total study population varied between 12% and 75%. An incidence of 75% was found in the article focusing on tumors in the opercular region.^35^ Meta-analysis on the incidence of neurological deficits shows a pooled incidence of 32% (95% CI: 23%-41%) (Figure 3A). Ten of 11 studies specified whether early postoperative deficits persisted.^13^^,^^21^^,^^25-27^^,^^29^^,^^33^^,^^35-37^ Deficits recovered in 8% to 49% of the total population, while 5% to 50% had persistent deficits. Three additional studies only quantified the occurrence of persistent deficits, which were 0%, 9%, and 38%.^24^^,^^30^^,^^38^

### Postoperative Ischemia and Neurological Deficits

Eleven studies in meta-analysis with 1064 patients reported presence of both postoperative ischemia on DWI and any postoperative neurological deficits.^13^^,^^23^^,^^25-27^^,^^29^^,^^32-35^^,^^37^ The combined incidence varied between 5% and 75%. The pooled incidence of a combination of postoperative ischemia and neurological deficits was 15% (95% CI: 10%-20%) (Figure 3B). Six studies specified whether early postoperative deficits recovered or persisted.^13^^,^^25^^,^^27^^,^^29^^,^^33^^,^^35^ A combination of postoperative ischemia and early postoperative neurological deficits were present in 10% to 75% of the population. Deficits in the presence of postoperative ischemia recovered in 3% to 25% of the total population, while 1% to 50% of patients had persistent deficits. Three additional studies reported both postoperative persistent neurological deficits and ischemia occurring in 0%, 5%, and 33% of patients.^23^^,^^30^^,^^38^ Within the group of patients with postoperative ischemia, new deficits were found in 14% to 75% with a pooled incidence of 41% (95% CI: 30%-52%) (Figure 3C).^13^^,^^23^^,^^25-27^^,^^29^^,^^32-35^^,^^37^ Six studies reported early postoperative deficits in 20% to 75% of the patients with postoperative ischemia. Deficits recovered in 5% to 67% of patients with ischemia and persisted in 8% to 50%. Three studies that only reported persistent deficits found those deficits in 0% and 47% of patients with ischemia.^23^^,^^30^^,^^38^ Conversely, the pooled incidence of patients with postoperative ischemia without observed deficits was 26% (95% CI: 17%-35%) (Figure 3D).^13^^,^^23^^,^^25-27^^,^^29^^,^^32-35^^,^^37^

In addition to a numerical association, 10 studies—one of which not included in the meta-analysis—found new postoperative deficits that were concordant with the location of diffusion restriction.^13^^,^^23^^,^^24^^,^^27^^,^^29^^,^^32-35^^,^^37^ This relation was present more often in persistent neurological deficits than transient deficits.

### Risk Factors

**Figure 4.**
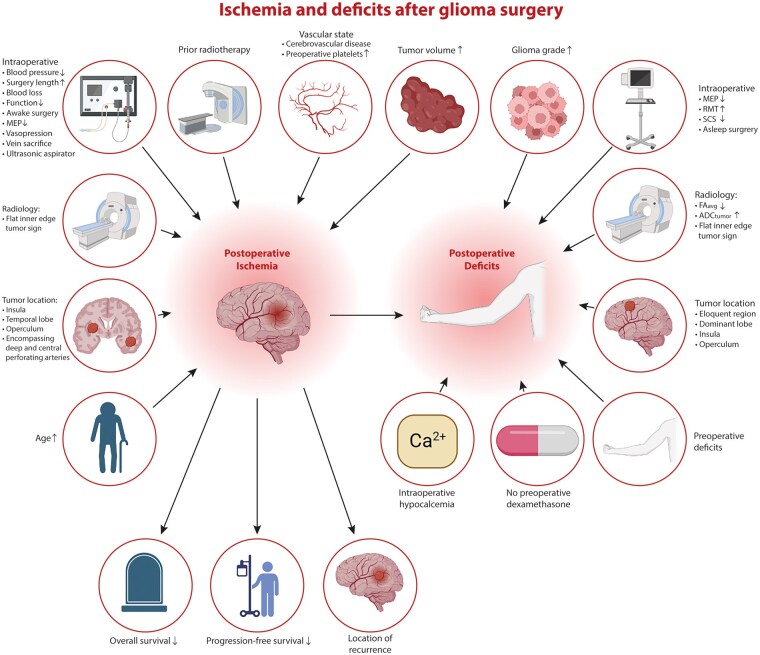
Summary of clinical, tumor, radiological, and intraoperative variables that influence and follow occurrence and extent of postoperative ischemia and new or worsened neurological deficits. Abbreviations: MEP: motor evoked potentials; RMT: resting motor threshold; SCS: subcortical stimulation; FA_avg_: average fractional anisotropy; ADC_tumor_: apparent diffusion coefficient in the tumor.

Potential risk factors for postoperative ischemia on DWI were reported in 12 of 29 studies (Table 3; Figure 4).^12^^,^^16^^,^^21-23^^,^^26^^,^^27^^,^^29^^,^^34^^,^^35^^,^^37^^,^^38^ Tumor location was associated with incidence of postoperative ischemia in 7 studies, identifying involvement of operculum, insula, temporal lobe, encapsulation by the tumor of deep perforating arteries, and proximity to central arteries as vulnerable locations.^12^^,^^23^^,^^27^^,^^29^^,^^34^^,^^35^^,^^37^ However, temporal tumor location was associated with absence of ischemia in our own cohort study.^27^ Furthermore, one study reported insular tumors as temporal localization.^12^ Other determinants were prior radiotherapy, preoperative platelet levels, a well-defined inner border of the tumor on MRI (flat inner edge sign), awake surgery, and perioperative decline of motor-evoked potentials or language function.^23^^,^^26^^,^^29^^,^^38^ Tumor volume, age, intraoperative mean diastolic and mean arterial blood pressure, length of surgery, fluid balance, and blood loss were associated with volume of postoperative infarctions.^12^^,^^16^ Presence of ischemia negatively impacted early functional performance scores, progression-free survival (PFS) and overall survival (OS), and was associated with diffuse and distant tumor recurrence in some studies.^14^^,^^15^^,^^20^^,^^23^ Postoperative ischemia was also related to the location of recurrence in one study (Figure 4).^18^

Presence of ischemia was associated with postoperative neurological deficits in 7 studies.^23-25^^,^^27^^,^^29^^,^^33^^,^^38^ Other determinants were high-grade glioma; preoperative deficits; encapsulation by the tumor of deep perforating arteries; infiltration into the dominant lobe, the insula, fronto-lateral operculum, parietal operculum, or other eloquent brain areas; radiological characteristics such as flat inner edge sign, lower average fractional anisotropy (FA), and higher apparent diffusion coefficient (ADC) in the tumor; asleep surgery; and adverse intraoperative neurophysiological characteristics including motor evoked potentials (MEP) decline, a higher resting motor threshold (RMT) in the tumor, and lower intensities of monopolar subcortical stimulation (SCS). Finally, no administration of preoperative dexamethasone was associated with early postoperative deficits and intraoperative hypocalcemia with severe (CTCAE grade ≥ 3) persistent deficits in our cohort (Table 3; Figure 4).^13^^,^^21^^,^^23-27^^,^^36^^,^^37^

## Discussion

The primary aim of this review was to explore the incidence of ischemia after neurosurgical glioma resection and its risk factors. The pooled incidence of postoperative ischemia is 48% when considering all tumor locations and 32% for new or worsened neurological deficits of any nature. We identified several risk factors for ischemia and deficits involving patient, tumor, and intraoperative characteristics, as summarized in Figure 4 and Table 3.

There was a large variation in incidence of ischemia and neurological deficits, which could be attributed to the heterogeneity of the included population. Definitions of postoperative ischemia also varied, as some studies included all grades of diffusion restriction,^14-16^ others included diffusion restriction of minimum size or certain type,^12^^,^^24^^,^^28^ and one study made use of maximum ADC values only.^11^ Furthermore, several studies excluded lesions of diffusion restriction related to methemoglobin.^26^^,^^29-32^

To avoid bias, only studies that performed DWI within 72 h after surgery were included for this review because diffusion restriction due to ischemia is thought to normalize after the acute phase.^39^ As a result, performing DWI within 72 h was standard of care in the centers that conducted the included studies. However, due to missing postoperative DWI within 72 h, 68 cases were excluded out of a potential 2788 (of which 5 were excluded by the authors), representing 2.4% of the total sample.^12^^,^^24^^,^^33-35^ Furthermore, 147 cases were excluded due to unspecified reasons, of which absence of postoperative DWI within 72 h could not be ruled out.^25^^,^^27^

The cause and course of postoperative ischemia is not yet fully understood. Pressure from surgical tools used for retraction of brain tissue may lead to reduced perfusion contusion of peritumoral tissue.^32^^,^^40^ Intraoperative hemodynamics play a role with a relation between larger postoperative infarct volumes and reduced intraoperative diastolic blood pressure, fluid balance, and a longer duration of surgery.^16^ In a previous study, we found an association between the use of perioperative vasopressive drugs and postoperative confluent ischemia as well.^27^

Anatomy also plays a role, as Kumabe et al.found postoperative ischemia in all 8 consecutive opercular glioma WHO grade II-IV patients with postoperative DWI.^35^ The afferent vessels in this region are all end arteries without substantial collaterals and, when at risk, result in high possibility of infarction. Other studies confirm this finding in the operculum and also report more postoperative ischemia in patients with insular and temporal tumors.^12^^,^^23^^,^^25^^,^^27^^,^^34^ In contrast, our group found less ischemia in tumors involving the temporal lobe.^27^ Although discrepancies could have been caused by a difference in surgical technique, different definitions of tumor locations have been utilized in analysis, as we analyzed involvement rather than predefined tumor locations, and Strand et al.included insular lesions in their definition of temporal tumors.^12^

Furthermore, tumor proximity to and encapsulation of deep perforating arteries were reported as significant risk factors for postoperative ischemia.^29^^,^^37^ Besides a more invasive approach to adequately resect these tumors, analyses of probability maps of postoperative ischemic lesions after glioma resection found a predominant risk near periventricular watershed areas, emphasizing the vulnerability of brain regions with a decreased or terminal blood supply.^12^ Since the development of postoperative ischemia appears to be linked to tumor location, inclusion of high-risk locations impacts the incidence ischemic lesions in this review. This is corroborated by a higher than average incidence of ischemia in the study involving the operculum.^35^ However, some studies reporting on insular tumors found less than the average pooled incidence of postoperative ischemia,^25^^,^^36^^,^^37^ while ischemic lesions were slightly more frequent in the study by Hou et al.^26^ As not all studies have reported the association between tumor location and postoperative ischemia, the overall spatial impact across cases in this review cannot be fully determined. Identification of vulnerable brain regions is important to assess vascular complications and future studies should include robust imaging to facilitate more precise spatial analyses.

Finally, awake surgery was associated with postoperative ischemia in a population of primary motor cortex gliomas, without increasing extent of resection.^38^ The authors attributed most of the ischemic lesions in their population to direct damage to perforating and passing arteries and some to vasospasms, suggesting an important role for tumor location and the decision for an awake procedure. Of note, one grade I glioma—excluded from the pooled incidence—was included in this analysis. Alternatively, the relation could be caused by a potential protective effect of sedation against transient ischemia.^41^ Of note, we did not find any association between the risk of ischemia and awake surgery in our surgical cohort.^27^

More ischemia was found after re-resection in a study in a heterogeneous population of patients including infratentorial and WHO grade I glioma.^42^ Gempt et al.elaborated on the possible causes, suggesting changes in vascularization due to development of scar tissue or as a result of therapeutic irradiation. However, Dützmann et al.did not find a significant difference in the presence of postoperative ischemia between first time and repetitive surgeries,^34^ despite using the same definition of ischemia as the former.

In a subsequent study, Gempt et al.correlated prior radiotherapy with postoperative ischemia.^29^ Although both studies report univariable results without correction for tumor grade, analyses were performed with a similar proportion of low- and high-grade tumors. This may indicate that the increased incidence of ischemia in the recurrent setting might have been due to radiotherapy. This is also in line with the damaging effect of radiation therapy on blood vessels and its potential role in the development of post-radiation injury.^43^ This was not confirmed in our or other cohorts, however.^27^

In recent years, the pathological field of gliomas has greatly changed. O(6)-methylguanine-DNA methyltransferase (MGMT) promoter methylation status drives prognosis and chemosensitivity in glioblastomas, but data on its relation with postoperative ischemia are limited.^6^ Some data suggest more ischemia with unmethylated GBM, but some imaging findings in unmethylated GBM may actually represent early progression rather than ischemia.^20^^,^^44^

New or worsened neurological deficits are frequent complications of postoperative ischemia. The pooled incidence of new or worsened postoperative neurological deficits was 32% in the whole population and even 42% in patients with postoperative ischemia, albeit with a large variance.^33^ Kumabe et al.found postoperative neurological deficits in 6 of 8 patients with opercular gliomas,^35^ and persistent neurological deficits in half of the population, clearly higher than the 6% to 38% reported in other studies.^13^^,^^21^^,^^25-27^^,^^29^^,^^30^^,^^33^^,^^35-38^ This could be explained by the reported high incidence of ischemia. The higher incidence of persistent motor deficits of 38% in the study by Magill et al.could very well be explained by the specific location of the tumors in the primary motor cortex.^38^ The role of ischemia can however not be excluded, as the authors report locally restricted diffusion in 5 of 6 cases with moderate to severe deficits. These latter findings were corroborated by Zetterling et al.who reported tumors in eloquent regions as a predictor of postoperative neurological deterioration, despite the use of surgery under awake circumstances or neuromonitoring.^13^ They found direct damage to the supplementary motor area and other eloquent areas, such as speech regions, to be the cause of most new, early postoperative neurological deficits. Delayed neurological deterioration was associated with postoperative seizures, however, illustrating that several factors besides ischemia may drive the occurrence of persisting neurological deficits. Of note, however, these analyses included 7 out of 100 cases that were excluded from the pooled incidence due to tumor types other than WHO grade II-IV gliomas. One study observed fewer transient motor deficits in patients who had an awake, transopercular resection of insular gliomas, compared to general anesthesia.^25^ This could very well be affected by tumor location, as tumors in the dominant hemisphere had a greater incidence of immediate deficits but also of awake resection. The comparable rate of persistent deficits between awake and asleep surgery might reflect a benefit of awake surgery in this group. Another study found more postoperative neurological deficits in patients with high-grade glioma, possibly due to increased risk of damage to surrounding vasculature and parenchyma during resection of highly invasive, poorly circumscribed lesions.^36^ Neurological deterioration is a potentially debilitating consequence of glioma surgery, as Berger et al.demonstrated a negative impact of ischemia on Karnofsky Performance Score (KPS) and modified Rankin Score (mRS) immediately after surgery.^23^ However, we did not find a difference in KPS between patients with and without postoperative ischemia in our own cohort at discharge or after 3 months.^27^ This was corroborated by Berger et al.in their analysis of functional outcome 6 months after surgery, showing either a recovery in the ischemic group or a delayed deterioration in the non-ischemic group.^23^

There was a clear connection between brain areas that were damaged by ischemia and the clinical symptoms of the patients.^32^ Gempt et al.found significantly more ischemic damage of the motor eloquent areas in patients with permanent motor deficits.^29^ New or worsened persistent deficits were concordant with the location of diffusion restriction in 21 of 27 patients in our cohort as well.^27^ A study by Neuloh et al. which was excluded from the review due to incomplete description of which patients received DWI, also reported deep ischemic lesions in the corona radiata in all 10 patients with persistent postoperative neurological deficits, without any direct surgical damage to the motor tract.^45^ In contrast, none of their patients with transient neurological deficits had ischemic lesions in the corticospinal tract. Biswas et al.observed a higher rate of persistent deficits in patients with infarcts concerning the lenticulostriate arteries as opposed to infarcts of the cortical or opercular branches of the middle cerebral artery.^25^ Finally, Rossi et al.reported DWI abnormalities along eloquent language tracts in patients with language deficits.^37^ However, as ischemic lesions most often occur along the border of the resection cavity, it might be hard to rule out direct damage or even edema as causes for new or worsened deficits. In fact, ischemia does not always have direct clinical consequences, as demonstrated by our finding that 26% of patients had postoperative ischemia without any new or worsened neurological deficits. Nevertheless, in cases of unexpected new neurological symptoms, ischemia should be considered a culprit when its location corresponds with the loss of function, especially when the symptoms were absent during resection in the awake setting. As the location of ischemia is essential for the nature of the clinical consequences, future studies should relate the clinical deficits of the patient to the presumed function of the damaged area as well as its potential for plasticity.

Besides neurological deterioration, postoperative ischemia can have other adverse consequences. Bette et al.showed that a larger postoperative infarct volume is an independent prognostic factor for poorer survival, likely through multifocal progression patterns, and discuss that hypoxic tissue might increase tumor regrowth.^14^^,^^15^ Although the relation between infarct volume overall survival was independent of age and KPS, its interaction with adjuvant treatment in this population was not reported. Nevertheless, location and invasiveness of recurrence have been associated with previous postoperative ischemia in other studies.^18^^,^^20^ Therefore, tumor biology appears to be influenced by a hypoxic environment, as demonstrated by experimental models.^46^ In contrast, a theory for a protecting aspect of postoperative ischemia is that tumors might not recur in infarcted areas, due to the impaired blood flow of the region.^40^ This latter theory is not supported by the reported evidence, however, and not all studies found a relation between postoperative ischemia and overall survival or progression-free survival.^23^

Recent studies such as those of the RANO resect group highlight the benefit of reducing the tumor load as much as possible.^47^^,^^48^ These results suggest that a supramarginal resection might lead to a better overall survival. Although extent of resection was not reported to be associated with ischemic lesions in the included studies, benefits of more aggressive surgical approaches should be weighed against the risks. As demonstrated by its consequences, it is important to minimize postoperative ischemia. Predisposing factors, such as tumor location in vulnerable regions, patient characteristics like age, and a history of cerebrovascular disease and radiological parameters such as the flat inner tumor edge sign can highlight the risk of developing postoperative ischemia and warrant extra caution. Neuromonitoring is a useful tool to guide safe resection, but its function is mainly aimed at outcome rather than a warning system, as irreversible MEP decline is usually associated with neurological ­deterioration.^21^^,^^24^^,^^25^ Intraoperative preservation of the cerebral vasculature as well as adequate hemodynamic management—including blood pressure and blood loss—appear to be the most important factors to prevent the occurrence and extent of postoperative ischemia.^12^^,^^16^^,^^22^ Even minute drops in blood pressure may result in ischemic lesions, as suggested by the fact that use of perioperative vasopression, but not blood pressure, was associated with ischemia in our population.^27^

Regarding limitations, most included studies were of a retrospective nature, known to be prone to various biases. However, quality assessment showed a low risk of bias in 23 studies, of which 20 included for meta-analysis, and a moderate risk of bias in 6 studies, of which 5 were included for meta-analysis. Nevertheless, the studies were heterogeneous, leading to different definitions of ischemia and neurological deficits. Finally, despite our best efforts to exclude cohorts from the same centers in similar periods, a small overlap in the population that was included in this meta-analysis cannot be ruled out.

As the aim of the current review was to assess the incidence of ischemia and its relation to postoperative deficits, we did not search for articles reporting solely on postoperative deficits. As such, the incidence of postoperative neurological deficits we found (32%) is higher than reported in a review by Wong et al.(up to 20%).^49^ The higher incidence of deficits in our review could reflect a specific subpopulation of glioma patients, wherein the authors focused on subgroups with an inherently high risk of postoperative ischemia, deficits, or both. Alternatively, the focus of the studies in our review on ischemia and deficits may have provided a more precise, and thus higher, reporting of deficits than other studies. Indeed, an investigation of new or worsened deficits in the glioblastoma population showed an incidence of almost 30% when including also mild deficits.^50^

Risk factors were reported for the studies that investigated associations of postoperative ischemia or neurological deficits. However, we excluded some cases in a few studies for the pooled incidences, due to tumor types other than WHO grade II-IV gliomas^13^^,^^35^^,^^38^ or missing postoperative DWI within 72 h.^24^^,^^33^^,^^35^ These cases were included in the original association analyses of the studies, which may have introduced bias for our research question. However, due to the low number of excluded cases, influence on the results is unlikely. In fact, combining and comparing potential risk factors from multiple studies may strengthen their validity.

## Conclusion

Postoperative ischemia and neurological deficits, both transient and persisting, are quite frequent consequences of glioma surgery. Available literature describes a great variety around the pooled incidence of 48%. Although it ultimately remains unclear what the causes for ischemia are, potential risk factors identified in literature are certain brain and perivascular locations, tumor volume, radiological characteristics, patient age and history, prior radiotherapy, and (indication for) awake surgery. Intraoperative hemodynamics may influence both infarction incidence and volume. It is suspected that postoperative ischemia plays a role in the development of postoperative neurological deficits and may even lead to a more invasive tumor recurrence. More prospective research is needed to confirm these claims, to elucidate the cause of ischemia, and to further assess the influence of postoperative ischemia on postsurgical neurological deficits and the course of disease.

## Supplementary Material

npaf122_Supplementary_Data

## Data Availability

Data from the published literature were used exclusively in this study. All the literature is referenced in the manuscript.

## Registration Information

This review is registered in PROSPERO (ID CRD42025564728).
