# Supplementary material for: Postoperative ischemia and neurological deficits after glioma resection: A systematic review and meta-analysis
Source: Neurooncol Pract. 2025 Dec 16;13(3):452–64. doi: 10.1093/nop/npaf122 (PMC13161909; doi:10.1093/nop/npaf122)
Supplement: npaf122_Supplementary_Data [file npaf122_supplementary_data.zip › npaf122_Supplementary_Data/Glioma_ischemia_review_-_Supplementary_Materials.docx]

**Supplementary material**

Supplementary 1: Search strategy Page 2

Supplementary 2: Quality assessment according to the modified NOS Page 3

| **Pubmed** |
| --- |
| postoperative[Title/Abstract] OR post operative[Title/Abstract] OR postsurgical[Title/Abstract] OR post surgical[Title/Abstract] OR postsurgery[Title/Abstract] OR post surgery[Title/Abstract] OR surger*[Title/Abstract] OR neurosurger*[Title/Abstract] OR resection*[Title/Abstract] OR debulking*[Title/Abstract] OR ("Cytoreduction Surgical Procedures"[Mesh]) OR ("Neurosurgical Procedures"[Mesh])  **AND**  glioma*[Title/Abstract] OR glioblastoma*[Title/Abstract] OR gbm[Title/Abstract] OR brain tumor*[Title/Abstract] OR brain tumour*[Title/Abstract] OR ("Glioma"[Mesh]) OR ("Supratentorial Neoplasms"[Mesh:NoExp])  **AND**  ischemi*[Title/Abstract] OR ischaemi*[Title/Abstract] OR infarct*[Title/Abstract] OR diffusion restriction*[Title/Abstract] OR diffusion weighted[Title/Abstract] OR DWI[Title/Abstract] OR ("Brain Ischemia"[Mesh:NoExp]) OR ("Brain Infarction"[Mesh:NoExp]) OR ("Cerebral Infarction"[Mesh]) OR ("Diffusion Magnetic Resonance Imaging"[Mesh:NoExp])  01-07-2024 |
| **Embase** |
| postoperative:ti,ab,kw OR 'post operative':ti,ab,kw OR postsurgical:ti,ab,kw OR 'post surgical':ti,ab,kw OR postsurgery:ti,ab,kw OR 'post surgery':ti,ab,kw OR surger*:ti,ab,kw OR neurosurger*:ti,ab,kw OR resection*:ti,ab,kw OR debulking*:ti,ab,kw OR 'cytoreductive surgery'/exp OR 'neurosurgery'/exp  **AND**  glioma*:ti,ab,kw OR glioblastoma*:ti,ab,kw OR gbm:ti,ab,kw OR 'brain tumor*':ti,ab,kw OR 'brain tumour*':ti,ab,kw OR 'glioma'/exp OR 'brain tumor'/de OR 'brain cancer'/de OR 'intracranial tumor'/de  **AND**  ischemi*:ti,ab,kw OR ischaemi*:ti,ab,kw OR infarct*:ti,ab,kw OR 'diffusion restriction*':ti,ab,kw OR 'diffusion weighted':ti,ab,kw OR dwi:ti,ab,kw OR 'brain ischemia'/exp OR 'brain infarction'/exp OR 'diffusion weighted imaging'/exp  **AND**  [embase]/lim  **AND**  'article'/it OR 'article in press'/it OR 'review'/it  01-07-2024 |
| **Cochrane Library** |
| (Postoperative OR post operative OR postsurgical OR post surgical OR postsurgery OR post surgery OR surger* OR neurosurger* OR resection* OR debulking*):ti,ab,kw  **AND**  (Glioma* OR glioblastoma* OR GBM OR brain tumor* OR brain tumour*):ti,ab,kw  **AND**  (Ischemi* OR ischaemi* OR infarct* OR diffusion restriction* OR diffusion weighted OR DWI):ti,ab,kw  01-07-2024 |

**Supplementary 1. Search strategy**

| **Study** | **Selection** | | | **Outcome** | | | **Modified quality score** |
| --- | --- | --- | --- | --- | --- | --- | --- |
|  | Representativeness of exposed cohort | Ascertainment of exposure | Demonstration that the outcome of interest was not present at the start of the study | Ascertainment of outcome | Follow-up | Loss of follow-up |  |
| Smith (2005)^1^ | * | * | * | * | * | * | 6 |
| Ulmer (2006)^2^ | * | * |  | * | * | * | 5 |
| Kumabe (2007)^3^ |  | * | * | * | * | * | 5 |
| Pirzkal (2009)^4^ | * | * | * | * | * | * | 6 |
| Dützmann (2012)^5^ | * | * |  | * | * | * | 5 |
| Gempt^6^ (2013) |  | * | * | * | * | * | 5 |
| Farace (2013)^7^ | * | * | * | * | * |  | 5 |
| Pamir (2013)^8^ |  | * | * | * | * | * | 5 |
| Thiepold (2015)^9^ |  | * |  | * | * | * | 4 |
| Bette (2016)^10^ | * | * | * | * | * | * | 6 |
| Majós (2016)^11^ | * | * | * | * | * | * | 6 |
| Bette (2017)^12^  ^a^ | * | * |  | * | * | * | 5 |
| Bette (2018)^13^  ^a^ | * | * | * | * | * | * | 6 |
| Magill (2018)^14^ |  | * |  | * | * | * | 4 |
| Loit (2019)^15^ | * | * | * | * | * | * | 6 |
| Mandonnet (2019)^16^  ^a^ |  | * |  | * | * | * | 4 |
| White (2019)^17^ | * | * | * | * | * | * | 6 |
| Bø (2020)^18^ | * | * |  | * | * | * | 5 |
| Przybylowski (2020)^19^ |  | * | * | * | * | * | 5 |
| Zetterling (2020)^20^ | * | * |  | * | * | * | 5 |
| Rossi (2021)^21^ |  | * | * | * | * | * | 5 |
| Rosenstock (2021)^22^ |  | * |  | * | * | * | 4 |
| Strand (2021)^23^ | * | * | * | * | * | * | 6 |
| Hou (2022)^24^ |  | * |  | * | * | * | 4 |
| Strand (2022)^25^  ^a^ | * | * | * | * | * | * | 6 |
| Berger (2022)^26^ | * | * |  | * | * | * | 5 |
| Van der Boog (2023)^27^ | * | * | * | * | * | * | 6 |
| Morshed (2024)^28^ |  | * |  | * | * | * | 4 |
| Biswas (2024)^29^ |  | * | * | * | * | * | 5 |

Supplementary 2. Quality assessment according to the modified NOS. A star is awarded when the criterion is met.

^a^ These studies were not included for meta-analysis

**References**

1. Smith JS, Cha S, Mayo MC, et al. Serial diffusion-weighted magnetic resonance imaging in cases of glioma: distinguishing tumor recurrence from postresection injury. J Neurosurg. 2005;103:428-438.

2. Ulmer S, Braga TA, Barker FG, Lev MH, Gonzalez RG, Henson JW. Clinical and radiographic features of peritumoral infarction following resection of glioblastoma. Neurology. 2006;67:1668-1670.

3. Kumabe T, Higano S, Takahashi S, Tominaga T. Ischemic complications associated with resection of opercular glioma. J Neurosurg. 2007;106:263-269.

4. Pirzkall A, McGue C, Saraswathy S, et al. Tumor regrowth between surgery and initiation of adjuvant therapy in patients with newly diagnosed glioblastoma. Neuro Oncol. 2009;11:842-852.

5. Dützmann S, Geßler F, Bink A, et al. Risk of ischemia in glioma surgery: comparison of first and repeat procedures. J Neurooncol. 2012;107:599-607.

6. Gempt J, Krieg SM, Hüttinger S, et al. Postoperative ischemic changes after glioma resection identified by diffusion-weighted magnetic resonance imaging and their association with intraoperative motor evoked potentials: clinical article. J Neurosurg. 2013;119:829-836.

7. Farace P, Amelio D, Ricciardi GK, et al. Early MRI changes in glioblastoma in the period between surgery and adjuvant therapy. J Neurooncol.

8. Pamir MN, Özduman K, Yildiz E, Sav A, Dinçer A. Intraoperative magnetic resonance spectroscopy for identification of residual tumor during low-grade glioma surgery: clinical article. J Neurosurg. 2013;118:1191-1198.

9. Thiepold A-L, Luger S, Wagner M, et al. Perioperative cerebral ischemia promote infiltrative recurrence in glioblastoma. Oncotarget. 2015;6:14537-14544.

10. Bette S, Wiestler B, Kaesmacher J, et al. Infarct volume after glioblastoma surgery as an independent prognostic factor. Oncotarget. 2016;7:61945-61954.

11. Majós C, Cos M, Castañer S, et al. Early post-operative magnetic resonance imaging in glioblastoma: correlation among radiological findings and overall survival in 60 patients. Eur Radiol. 2016;26:1048-1055.

12. Bette S, Wiestler B, Wiedenmann F, et al. Safe brain tumor resection does not depend on surgery alone-role of hemodynamics. Sci Rep. 2017;7:5585-5588.

13. Bette S, Barz M, Huber T, et al. Retrospective analysis of radiological recurrence patterns in glioblastoma, their prognostic value and association to postoperative infarct volume. Sci Rep. 2018;8:4561.

14. Magill ST, Han SJ, Li J, Berger MS. Resection of primary motor cortex tumors: feasibility and surgical outcomes. J Neurosurg. 2018;129:961-972.

15. Loit M-P, Rheault F, Gayat E, et al. Hotspots of small strokes in glioma surgery: an overlooked risk? Acta Neurochir (Wien). 2019;161:91-98.

16. Mandonnet E. Transopercular resection of IDH-mutated insular glioma: a critical appraisal of an initial experience. World Neurosurg. 2019;132:e563-e576.

17. White ML, Zhang Y, Yu F, et al. Post-operative perfusion and diffusion MR imaging and tumor progression in high-grade gliomas. PLoS One. 2019;14:e021390.

18. Bø HK, Solheim O, Kvistad K-A, et al. Intraoperative 3D ultrasound–guided resection of diffuse low-grade gliomas: radiological and clinical results. J Neurosurg. 2020;132:518-529.

19. Przybylowski CJ, Baranoski JF, So VM, Wilson J, Sanai N. Surgical morbidity of transsylvian versus transcortical approaches to insular gliomas. J Neurosurg. 2020;132:1731-1738.

20. Zetterling M, Elf K, Semnic R, Latini F, Engström ER. Time course of neurological deficits after surgery for primary brain tumours. Acta Neurochir (Wien). 2020;162:3005-3018.

21. Rossi M, Gay L, Conti Nibali M, et al. Challenging giant insular gliomas with brain mapping: evaluation of neurosurgical, neurological, neuropsychological, and quality of life results in a large mono-institutional series. Front Oncol. 2021;11:629166.

22. Rosenstock T, Tuncer MS, Münch MR, Vajkoczy P, Picht T, Faust K. Preoperative nTMS and intraoperative neurophysiology—a comparative analysis in patients with motor-eloquent glioma. Front Oncol. 2021;11:676626.

23. Strand PS, Berntsen EM, Fyllingen EH, et al. Brain infarctions after glioma surgery: prevalence, radiological characteristics and risk factors. Acta Neurochir (Wien)). 2021;163:3097-3108.

24. Hou Z, Huang Z, Li Z, et al. Incidence of ischemic complications and technical nuances of arteries preservation for insular gliomas resection. Front Surg. 2022;9:956872.

25. Strand PS, Gulati S, Sagberg LM, Solheim O. Intraoperative risk factors for peritumoral infarctions following glioma surgery. Brain and Spine. 2022;2:100903.

26. Berger A, Tzarfati GG, Serafimova M, et al. Risk factors and prognostic implications of surgery-related strokes following resection of high-grade glioma. Sci Rep. 2022;12:22594.

27. van der Boog ATJ, Rados M, Akkermans A, et al. Occurrence, risk factors, and consequences of postoperative ischemia after glioma resection: a retrospective study. Neurosurgery. 2023;92:125-136.

28. Morshed RA, Cummins DD, Clark JP, et al. Asleep triple-modality motor mapping for perirolandic gliomas: an update on outcomes. J Neurosurg. 2024;140:1029-1037.

29. Biswas C, Shetty PM, Sahu A, et al. Factors affecting the extent of resection and neurological outcomes following transopercular resection of insular gliomas. Acta Neurochir (Wien). 2024;166:244.
