# Supplementary figures and images for: Postoperative ischemia and neurological deficits after glioma resection: A systematic review and meta-analysis
Source: Neurooncol Pract. 2025 Dec 16;13(3):452–64. doi: 10.1093/nop/npaf122 (PMC13161909; doi:10.1093/nop/npaf122)

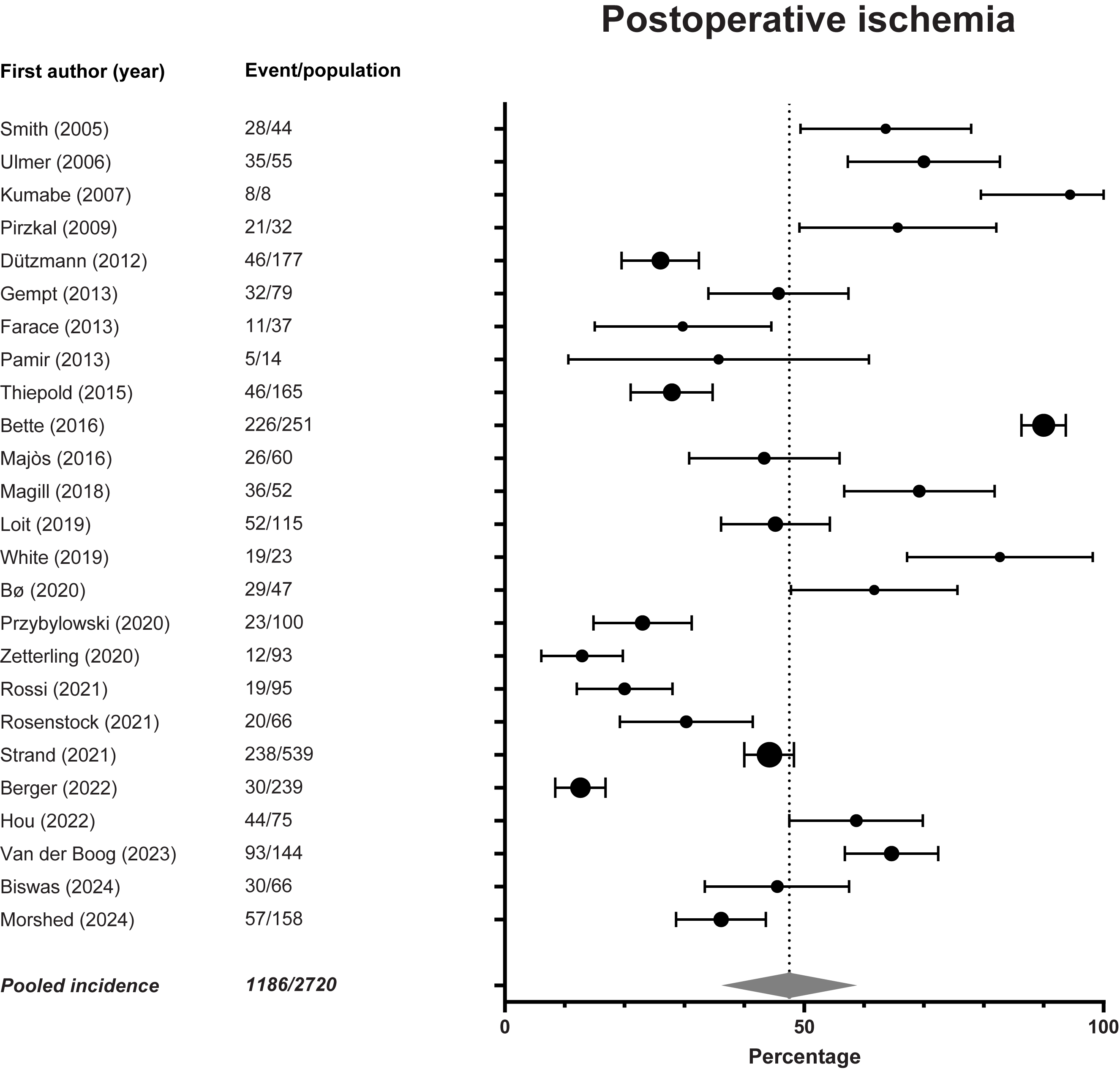

Supplement: npaf122_Supplementary_Data [file npaf122_supplementary_data.zip › npaf122_Supplementary_Data/Ischemia review - Figure 2.tif]

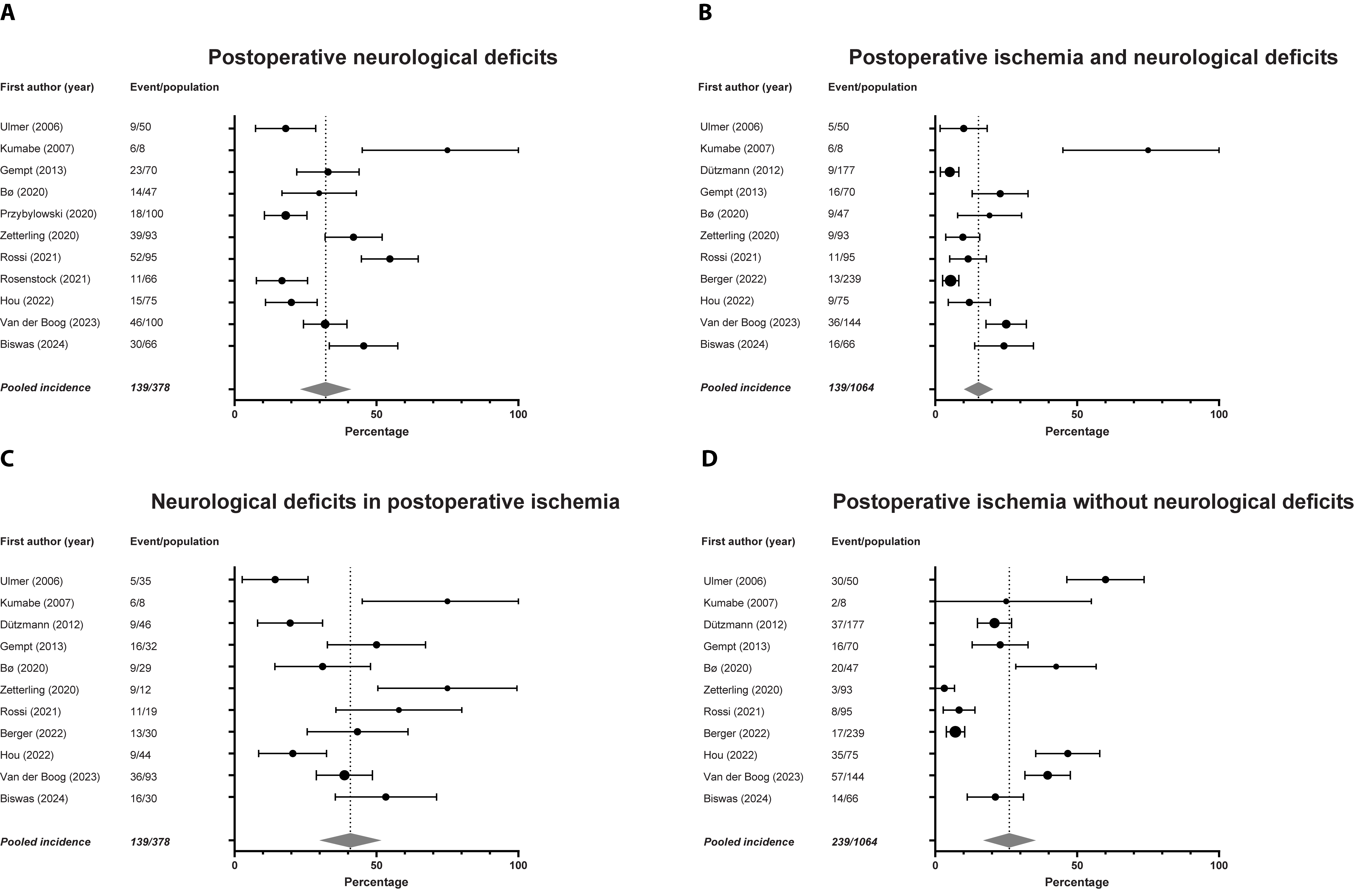

Supplement: npaf122_Supplementary_Data [file npaf122_supplementary_data.zip › npaf122_Supplementary_Data/Ischemia review - Figure 3.tif]
