# Supplementary material for: Postoperative ischemia and neurological deficits after glioma resection: A systematic review and meta-analysis
Source: Neurooncol Pract. 2025 Dec 16;13(3):452–64. doi: 10.1093/nop/npaf122 (PMC13161909; doi:10.1093/nop/npaf122)
Supplement: npaf122_Supplementary_Data [file npaf122_supplementary_data.zip › npaf122_Supplementary_Data/Glioma_ischemia_review_-_Tables_Rebuttal.docx]

| **Study** | **Study design** | **Population size (n)** | **Tumor** | **Tumor location** | **Tumor volume (cm^3^)** | **MRI field strength (Tesla)** | **Time to DWI (h)** | **Recurrence (n)** | **Prior radiotherapy** |
| --- | --- | --- | --- | --- | --- | --- | --- | --- | --- |
| Smith (2005)^11^ | Prospective | 44 | Glioma WHO grade II-IV | All lobes | ·· | 1.5 | <48 | ·· | ·· |
| Ulmer (2006)^32^ | Retrospective | 50 | GBM | Unspecified | ·· | 1.5 | <72 | ·· | ·· |
| Kumabe (2007)^35^ | Retrospective | 8 | Glioma WHO grade II-IV | Operculum (around orofacial cortices) | ·· | 1.5 | <72 | ·· | ·· |
| Pirzkal (2009)^17^ | Retrospective | 32 | GBM | Unspecified | ·· | 1.5 | Immediately | 0 | 0 |
| Dützmann (2012)^34^ | Retrospective | 177 | Glioma WHO grade II-IV | All lobes | ·· | 1.5 | <72 | 47 | ·· |
| Gempt (2013)^29^ | Retrospective | 70 | Glioma WHO grade II-IV | All lobes | ·· | 3 | <48 | 19 | 10 |
| Farace (2013)^19^ | Retrospective | 37 | GBM | Unspecified | ·· | 1 | <72 | ·· | ·· |
| Pamir (2013)^10^ | Prospective | 14 | Glioma WHO grade II | All lobes | ·· | 3 | 24-48 | ·· | ·· |
| Thiepold (2015)^20^ | Retrospective | 165 | GBM | Unspecified | ·· | 3 | <72 | ·· | ·· |
| Bette (2016)^15^ | Retrospective | 251 | GBM | Unspecified | 30.7 | 3 | Unspecified | 0 | 1 |
| Majós (2016)^28^ | Retrospective | 60 | GBM | Unspecified | ·· | 1.5 | <72 | ·· | ·· |
| Bette (2017)^16^ | Retrospective | 179 | GBM | Unspecified | 24.6 | 3 | Unspecified | 44 | 42 |
| Bette (2018)^14^ | Retrospective | 129 | GBM | Unspecified | 29.6 | 3 | <72 | 0 | 0 |
| Magill (2018)^38^ | Retrospective | 52 | Glioma WHO grade II-IV | Primary motor cortex | ·· | Unspecified | <48h | 4 | ·· |
| Loit (2019)^30^ | Retrospective | 115 | Glioma WHO grade II-IV | All lobes | ·· | 1.5 / 3 | <48 | ·· | ·· |
| Mandonnet (2019)^31^ | Retrospective | 12 | IDH-mutated glioma | Insula | 68.5 | Unspecified | Immediately | 0 | 0 |
| White (2019)^18^ | Retrospective | 23 | High grade glioma | Unspecified | ·· | 1.5 / 3 | <24 | ·· | ·· |
| Bø (2020)^33^ | Retrospective | 47 | Glioma WHO grade II | All lobes | 23.2 | 1.5 / 3 | <72 | 0 | 0 |
| Przybylowski (2020)^36^ | Retrospective | 100 | Glioma WHO grade II-IV | Insula | 62.4 | Unspecified | <48 | 18 | ·· |
| Zetterling (2020)^13^ | Prospective | 93 | Glioma WHO grade II-IV | All lobes | ·· | Unspecified | <48 | ·· | ·· |
| Rossi (2021)^37^ | Retrospective | 95 | Glioma WHO grade II-IV | Insula | 71.1 | 3 | <48 | 45 | ·· |
| Rosenstock (2021)^21^ | Retrospective | 66 | Glioma WHO grade II-IV | Motor eloquent regions | 23.4 | 3 | <72 | 22 | ·· |
| Strand (2021)^12^ | Prospective | 539 | Glioma WHO grade II-IV | All lobes | ·· | Unspecified | <72 | 219 | 140 |
| Berger (2022)^23^ | Retrospective | 239 | High grade glioma | All lobes | 32.8 | Unspecified | <48 | 86 | 69 |
| Hou (2022)^26^ | Retrospective | 75 | Glioma WHO grade II-IV | Insula | 57.3 | Unspecified | <72 | 2 | 0 |
| Strand (2022)^22^ | Retrospective | 175 | Glioma WHO grade II-IV | All lobes | ·· | Unspecified | <72 | 58 | ·· |
| Van der Boog (2023)^27^ | Retrospective | 144 | Glioma WHO grade II-IV | Unspecified | 33.2 | 1.5 | <72 | 36 | 28 |
| Morshed (2024)^24^ | Retrospective | 151 | Glioma WHO grade II-IV | Perirolandic | ·· | Unspecified | <48 | 68 | ·· |
| Biswas (2024)^25^ | Retrospective | 66 | Glioma WHO grade II-IV | Insula | 92.6 | Unspecified | <48 | 14 | 4 |

**Table 1. Baseline table of all included studies.**

| **Study** | **Population size (n) included in meta-analysis** | **Postoperative ischemia on DWI** | | **Ischemia volume (cm^3^)** | **Postoperative neurological deficits (n)** | | | **Combined ischemia and neurological deficits (n)** | | |
| --- | --- | --- | --- | --- | --- | --- | --- | --- | --- | --- |
|  |  | *n* | *%* |  | *Total* | *Transient* | *Persisting* | *Total* | *Transient* | *Persisting* |
| Smith (2005)^11^ | 44 | 28 | 63.6 | ·· | ·· | ·· | ·· | ·· | ·· | ·· |
| Ulmer (2006)^32^ | 50 | 35 | 70.0 | 14.9 | 9 | ·· | ·· | 5 | ·· | ·· |
| Kumabe (2007)^35^ | 8 | 8 | 100.0 | ·· | 6 | 2 | 4 | 6 | 2 | 4 |
| Pirzkal (2009)^17^ | 32 | 21 | 65.6 | ·· | ·· | ·· | ·· | ·· | ·· | ·· |
| Dützmann (2012)^34^ | 177 | 46 | 26.0 | ·· | ·· | ·· | ·· | 9 | ·· | ·· |
| Gempt (2013)^29^ | 70 | 32 | 45.7 | ·· | 23 | 13 | 10 | 16 | 8 | 8 |
| Farace (2013)^19^ | 37 | 11 | 29.7 | ·· | ·· | ·· | ·· | ·· | ·· | ·· |
| Pamir (2013)^10^ | 14 | 5 | 35.7 | ·· | ·· | ·· | ·· | ·· | ·· | ·· |
| Thiepold (2015)^20^ | 165 | 46 | 27.9 | 17.1 | ·· | ·· | ·· | ·· | ·· | ·· |
| Bette (2016)^15^ | 251 | 226 | 90.0 | 2.0 | ·· | ·· | ·· | ·· | ·· | ·· |
| Majós (2016)^28^ | 60 | 26 | 43.3 | ·· | ·· | ·· | ·· | ·· | ·· | ·· |
| Magill (2018)^38^ | 52 | 36 | 69.2 | ·· | ·· | ·· | 20 | ·· | ·· | 17 |
| Loit (2019)^30^ | 115 | 52 | 45.2 | 1.6 | ·· | ·· | 0* | ·· | ·· | 0^a^ |
| White (2019)^18^ | 23 | 19 | 82.6 | ·· | ·· | ·· | ·· | ·· | ·· | ·· |
| Bø (2020)^33^ | 47 | 29 | 61.7 | ·· | 14 | 10 | 4 | 9 | 7 | 2 |
| Przybylowski (2020)^36^ | 100 | 23 | 23.0 | ·· | 18 | 8 | 10 | ·· | ·· | ·· |
| Zetterling (2020)^13^ | 93 | 12 | 12.9 | ·· | 39 | 33 | 6 | 9 | 8 | 1 |
| Rossi (2021)^37^ | 95 | 19 | 20.0 | ·· | 52 | 47 | 5 | 11 | ·· | ·· |
| Rosenstock (2021)^21^ | 66 | 20 | 30.3 | ·· | 11 | ·· | 9^b^ | ·· | ·· | ·· |
| Strand (2021)^12^ | 539 | 238 | 44.2 | 1.7 | ·· | ·· | ·· | ·· | ·· | ·· |
| Berger (2022)^23^ | 239 | 30 | 12.6 | 13.2 | ·· | ·· | ·· | 13 | ·· | ·· |
| Hou (2022)^26^ | 75 | 44 | 58.7 | 7.8 | 9^c^ | 2 | 7 | 9 | 2 | 7 |
| Van der Boog (2023)^27^ | 144 | 93 | 64.6 | 3.5 | 46 | 11 | 27^d^ | 36 | 8 | 22^d^ |
| Morshed (2024)^24^ | 158 | 57 | 35.6 | ·· | ·· | ·· | 5 | ·· | ·· | 3 |
| Biswas (2024)^25^ | 66 | 30 | 45.5 | ·· | 30 | 20 | 7^e^ | 16 | 10 | 6 |

**Table 2. Incidence of postoperative ischemia and neurological deficits in the studies included in meta-analysis.**

^a^ only motor and language function, visual field defects were not taken into account

^b^ 61 patients available for 3 month follow-up

^c^ Only motor deficits taken into account

^d^ 129 patients available for 3 month follow-up; 2 deficits were identified at 3 month follow-up that were not reported at discharge

^e^ 27 patients available for 6 month follow-up

| **Study** | **Risk factors for postoperative ischemia** | **Risk factors for postoperative neurological deficits** |
| --- | --- | --- |
| Kumabe (2007)^35^ | Opercular tumor location  Posterior insular tumor location | ·· |
| Dützmann (2012)^34^ | Opercular tumor location  Insular tumor location  Temporal tumor location | ·· |
| Gempt (2013)^29^ | Proximity to central arteries  Prior radiotherapy | Ischemia |
| Bette (2016)^15^ | Mean peroperative diastolic blood pressure^a^  Peroperative arterial pressure^a^  Length of surgery^a^  Fluid balance^a^ | ·· |
| Magill (2018)^38^ | Awake surgical resection | Ischemia |
| Bø (2020)^33^ | ·· | Ischemia |
| Przybylowski (2020)^36^ | ·· | High grade glioma |
| Zetterling (2020)^13^ | ·· | Pre-operative deficits  Eloquent tumor location |
| Rossi (2021)^37^ | Tumor encapsulation of deep perforating arteries | Tumor encapsulation of deep perforating arteries |
| Rosenstock (2021)^21^ | ·· | Motor cortex infiltration  nTMS risk factors  Lower FA and higher ADC values  Irreversible MEP amplitude decrease |
| Strand (2021)^12^ | Temporal tumor location  Cerebrovascular disease^b^  Tumor volume^a^  Intra-operative bleeding^a^  Age | ·· |
| Berger (2022)^23^ | Insular tumor location  Temporal tumor location  Pre-operative high level of platelets  Per-operative language decline | Ischemia |
| Hou (2022)^26^ | MEP amplitude decline  Flat inner edge tumor sign | Flat inner edge tumor sign  MEP amplitude decline |
| Strand (2022)^22^ | Surgeon-reported vein sacrifice  Use of ultrasonic aspirator^b^ | ·· |
| Van der Boog (2023)^27^ | Insular tumor involvement  No temporal tumor involvement  Use of intraoperative vasopression | Confluent ischemia  Pre-operative dexamethasone  Intraoperative hypocalcemia^c^ |
| Morshed (2024)^24^ | ·· | Insular tumor location  Pre-operative deficits  Ischemia^d^  Intra-operative MEP changes |
| Biswas (2024)^25^ | ·· | Dominant lobe  Fronto-lateral opercular tumor location  Pariëtal opercular tumor location  Asleep surgery^e^  Ischemia^d^  MEP changes^d^ |

**Table 3. Risk factors for postoperative ischemia and neurological deficits.**

^a^ Related to volume of postoperative ischemia

^b^ Related to sector-shaped infarction

^c^ Related to severe deficits

^d^ Related to prolonged and/or persistent deficits

^e^ Related to transient deficits
